# Alternative Splicing for Diseases, Cancers, Drugs, and Databases

**DOI:** 10.1155/2013/703568

**Published:** 2013-05-22

**Authors:** Jen-Yang Tang, Jin-Ching Lee, Ming-Feng Hou, Chun-Lin Wang, Chien-Chi Chen, Hurng-Wern Huang, Hsueh-Wei Chang

**Affiliations:** ^1^Department of Radiation Oncology, Faculty of Medicine, College of Medicine, Kaohsiung Medical University, Kaohsiung 807, Taiwan; ^2^Department of Radiation Oncology, Kaohsiung Medical University Hospital, Kaohsiung 807, Taiwan; ^3^Cancer Center, Kaohsiung Medical University Hospital, Kaohsiung Medical University, Kaohsiung 807, Taiwan; ^4^Department of Biotechnology, Kaohsiung Medical University, Kaohsiung 807, Taiwan; ^5^Institute of Clinical Medicine, Kaohsiung Medical University, Kaohsiung 807, Taiwan; ^6^Kaohsiung Municipal Ta-Tung Hospital, Kaohsiung 807, Taiwan; ^7^Bioresource Collection and Research Center, Food Industry Research and Development Institute, Hsinchu 300, Taiwan; ^8^Institute of Biomedical Science, National Sun Yat-Sen University, Kaohsiung 807, Taiwan; ^9^Graduate Institute of Natural Products, Kaohsiung Medical University, Kaohsiung 807, Taiwan; ^10^Department of Biomedical Science and Environmental Biology, Kaohsiung Medical University, Kaohsiung 807, Taiwan

## Abstract

Alternative splicing is a major diversification mechanism in the human transcriptome and proteome. Several diseases, including cancers, have been associated with dysregulation of alternative splicing. Thus, correcting alternative splicing may restore normal cell physiology in patients with these diseases. This paper summarizes several alternative splicing-related diseases, including cancers and their target genes. Since new cancer drugs often target spliceosomes, several clinical drugs and natural products or their synthesized derivatives were analyzed to determine their effects on alternative splicing. Other agents known to have modulating effects on alternative splicing during therapeutic treatment of cancer are also discussed. Several commonly used bioinformatics resources are also summarized.

## 1. Introduction to Alternative Splicing

Alternative splicing of RNA is a key mechanism of increasing complexity in mRNA and proteins [1]. Since alternative splicing apparently controls almost all human gene activities, imbalances in the this splicing process may affect the progression of various human diseases and cancers [2]. Varying alternations in excision and/or inclusion of exons may generate different mRNA transcripts and corresponding proteins. Therefore, in addition to mediating changes in protein structure, function, and localization [3], alternative splicing in higher eukaryotes affects the differentiation and development of cancer and other diseases [4].

## 2. Alternative Splicing and Diseases

Alternative RNA splicing is commonly reported in neurological and muscle diseases [5–7]. Studies show that these diseases at least partly result from alternative splicing, which regulates the complexity of integral membrane proteins, including changes in their topology, solubility, and signal peptides [3]. For example, aberrant alternative splicing has shown associations with Parkinson disease [3, 8]. For spinal muscular atrophy (SMA), the level of survival motor neuron (SMN) protein was downregulated by its alternative splicing [9]. Therapies for SMA have recently improved by targeting RNA splicing for inclusion of exon 7 into SMN mRNA [10]. Phorbol 12-myristate 13-acetate was reported to modulate the alternative splicing of sarcoplasmic reticulum Ca^2+^-ATPase1 (SERCA1) which is dysregulated in myotonic dystrophy type 1 disease [11].

Additionally, alternative splicing reportedly regulates heart development [12], cardiovascular disease [13], blood coagulation [14], cholesterol homeostasis [15], cellular proliferation, apoptosis, immunity [16], and systemic sclerosis [17]. For example, heart-specific knockout of the serine/arginine- (SR-) rich family of splicing factors, ASF/SF2, produces cardiomyopathy and affects splicing of cardiac troponin T and LIM domain-binding protein [18]. Specific CLK inhibitors (dichloroindolyl enaminonitrile KH-CB19) of CDC2-like kinase isoforms 1 and 4 (CLK1/CLK4) can inhibit phosphorylation of cellular SR splicing factors and affect the splicing of tissue factor isoforms flTF (full-length TF) and asHTF (alternatively spliced human TF) [19].

Using exon array, the global mRNA splicing profile of ischemic cardiomyopathy has been investigated, and the alternative splicing of four main sarcomere genes, such as cardiac troponin T (TNNT2), cardiac troponin I (TNNI3), myosin heavy chain 7 (MYH7), and filamin C, gamma (FLNC), was dysregulated [20]. The alternative splicing of blood coagulation-related genes including tissue factor (coagulation factor III), tissue factor pathway inhibitor (TFPI), and coagulation factor XI has been well reviewed [14]. For cholesterol production and uptake, alternative splicing of 3-hydroxy-3-methylglutaryl coenzyme A reductase (HMGCR) and LDL receptor (LDLR) can suppress their protein activities [21, 22]. Proprotein convertase subtilisin/kexin type 9 (PCSK9) [23], HMG-CoA synthase (HMGCS1) [24], and mevalonate kinase [25] also reported to be involved in cholesterol biosynthesis and receptor-mediated uptake through alternative splicing.

## 3. Alternative Splicing and Cancer

In cancer-associated genes, splicing has important roles in oncogenesis, tumor suppression [26], and metastasis [27]. Alterations in alternative splicing are commonly reported in various cancers [27–29]. Reported examples include p53 and PTEN [30], kallikrein-related peptidase 12 (KLK12) [31], breast cancer early-onset 1 (BRCA1) [32], protein N-arginine methyltransferases 2 (PRMT2) [33], and CDC25 phosphatases [34] in breast cancer; lysyl oxidase-like 4 (LOXL4) [35] and growth factor receptor-bound protein 7 (GRB7) in ovarian cancer [36]; androgen receptor in prostate cancer [37]; tissue inhibitor of metalloproteinases-1 (TIMP1) and the cell adhesion molecule CD44 in colon cancer [38, 39]; Bcl-xL, CD44, and others in lung cancer [40]; calpain 3 in melanoma [41]; and Krüppel-like factor 6 (KLF6) in liver cancer [42]. Therefore, alternative spliced variants are potential biomarkers [43, 44] for the cancer diagnosis/prognosis and may be the targets for cancer therapy based on specific splicing correction treatments.

Single nucleotide polymorphisms (SNPs) affecting exon skipping has reviewed to confer to complex diseases [45]. The improvement of high-throughput technologies such as RNA-Seq [46, 47] and exon arrays [48, 49] is helpful to identify the genome-wide cancer-associated splicing variants. Splicing changes may associate with lung and prostate cancer risk in terms of some SNPs. For example, a coding synonymous SNP G870A of cyclin D1 (CCND1) with a modulating ability to its splice pattern was reported to be associated with lung cancer susceptibility [50]. Similarly, some coding synonymous SNPs may generate new splicing sites in the middle of an exon of p53 gene to change splicing [51]. Mutations in the adenomatous polyposis coli (APC) [52] and BRCA1 [53, 54] genes have reported to skip exon by altering splicing. Furthermore, an intronic SNP, IVS −27 G>A/IVSΔA, creates a new splicing factor SR-binding site and deletes two other overlapping SR-binding sites, generating three alternative splicing forms of KLF6 (KLF6 SV1-3) [55]. This SNP was found to be associated with prostate cancer [56]. For lung cancer study, the tumor patients with overexpression of KLF6 SV1 have lower survival [56, 57].

Actually, the information of many SNPs located in 3′ and 5′ splicing sites is searchable in the dbSNP in NCBI website (http://www.ncbi.nlm.nih.gov/snp) when the “limit” function is chosen. Researchers may choose the SNPs of splicing sites located in interested genes to identify their association relationships to diseases and cancers. A database consisting of genome-wide SNP and splicing sites, namely, ssSNPTarget [58] was designed to search the splice site SNPs (ssSNPs) by input of gene symbol, SNP rs number, transcript ID, or genomic position (http://variome.kobic.re.kr/ssSNPTarget/).

## 4. Alternative Splicing-Related Drugs and Natural Products

Splice modulating therapies have been developed for human disease [59–61] and cancer therapy [62, 63]. Antitumor drugs have been developed to target alternative splicing [64], splice variants [65], and spliceosomes [66, 67]. For example, pharmacological interventions may be affected by mRNA transcript diversity [68]. To correct aberrant splicing, specific mRNA transcripts have been targeted in genetic disorders such as Duchenne muscular dystrophy. Since mutations of splicing factor 3B subunit 1 (SF3B1) are common in several haematological malignancies, the use of various natural products and their synthetic derivatives in therapies targeting SF3B has proven highly effective [67].

For drug discovery for SMA, several small molecules including sodium vanadate [69], aclarubicin [70], and indoprofen [71], hydroxyurea [72], valproate [73], 5-(N-ethyl-N-isopropyl)-amiloride (EIPA) [74], and phenylbutyrate [75] that increases inclusion of exon 7 of SMN2 gene have been identified, although some of them may have side effects. Recently, novel phosphatase modulators, namely, pseudocantharidins have been discovered with the similar regulating function to SMN splicing [76]. Valproic acid was found to enhance SMN2 expression in SMA cell model involving the SF2/ASF and hnRNPA1 [77].

Clinical drugs such as novantrone (mitoxantrone) can enhance the effectiveness of therapeutic treatments for familial neurodegenerative diseases by stabilizing the tau pre-mRNA splicing regulatory element [78]. Tamoxifen has proven effective for clinical treatment of estrogen receptor- (ER-) positive breast cancer [79]. In endometrial cancer cells, alternative splicing of ER involving ER-alpha36 is also known to enhance the agonist activity of tamoxifen [80].

Natural products, including many xenobiotics, are also known to impair alternative splicing [81]. For example, natural products such as pladienolide B and FR901464 [82, 83] are known to affect spliceosome function. However, the synthesis of these compounds is complicated by their multiple stereocenters. A recent study synthesized Sudemycins, which are novel analogues of FR901464. By inducing alternative gene splicing, the Sudemycins conferred both *in vitro *and *in vivo* antitumor effects [84]. Alternative splicing has also shown regulating effects on the antitumor drug Spliceostatin A, a stabilized derivative of a *Pseudomonas* bacterial fermentation product [85] which specifically targets the SF3b spliceosome subcomplex to inhibit pre-mRNA splicing [86]. Meayamycin, an analogue of the natural antitumor product FR901464 [87], inhibits RNA splicing against multidrug-resistant cells and performs antiproliferative effect against human breast cancer MCF-7 cells by suppression of alternative splicing [88]. These results suggest that, because of their modulating effects on RNA splicing, xenobiotic analogs have potential use as chemical probes and as anticancer agents. Similarly, the polyketide natural product borrelidin inhibits cancer metastasis by modulating alternative splicing in VEGF [89]. Antitumor effects involving alternative splicing [90, 91] have also been reported in natural dietary products. For example, resveratrol can modulate exon inclusion of SRp20 and SMN2 pre-mRNAs and induce the expression of processing factors of alternative splicing such as ASF/SF2, hnRNPA1, and HuR [92].

Recent studies have investigated the role of splice variants in apoptotic pathways [93, 94]. Regulation of alternative splicing genes may have anticancer effects. For example, BCL-X_s_ and BCL-X_L_ have been associated with proapoptotic and antiapoptotic effects, respectively, during the progression of cancer [67, 95, 96]. The ratio of BCL-X_s_/BCL-X_L_ can be decreased by the treatment of protein kinase C (PKC) inhibitor and apoptotic inducer staurosporine in 293 cells [97]. Soluble and membrane-bound forms of TNF receptor superfamily, member 6 (FAS) containing exons 5/7 and 5/6/7 also display proapoptotic and antiapoptotic effects, respectively [67]. The caspase 9 (CASP9) gene has two antagonistic isoforms, proapoptotic Casp9a and prosurvival Casp9b, and its splicing is dysregulated in NSCLC lung cancer cell lines [98].

Alternative splicing is regulated by chromatin structure and histone modifications [4]. In thyroid tumor cells, for example, histone deacetylase inhibitors such as butyrate modulate transcription and alternative splicing of prohibitin [99]. A study of bovine epithelial cells showed that butyrate, a major metabolite generated by bacterial fermentation of dietary fiber in colon cells, has regulating effects on apoptosis and cell proliferation through alternative splicing [100]. Since histone deacetylase inhibitor may have antitumor effects, the identification of this kind of inhibitor in natural products can improve drug development for tumor therapy. 

## 5. Alternative Splicing-Related Bioinformatics Resources

Several bioinformatics analyses for the detection and regulation of alternative splicing have been well reviewed [101–103]. However, these literatures mainly focused on the methodology for detection of alternative splicing, and the databases of alternative splicing are less addressed and summarized. Here, we collect several helpful bioinformatics resources related to alternative splicing as shown in Table 1.

 For example, AsMamDB [104] is one of the early established alternative splice databases of mammals, although their websites are not functional currently. PALS db [105] provides the putative alternative splicing database based on UniGene clusters of human and mouse sources which mainly consist of EST data. Similarly, some databases such as EASED [107] and AVATAR [108] are constructed by datasets of EST and mRNAs. ASAP [106] provides the detail annotation for exon-intron boundary, alternative splicing, and its tissue specificity for the user to design probes for distinguishing different splicing isoforms. MAASE [109] is also specifically designed to apply in splicing microarray experiments. In contrast, Splicy [115] provide the web-based tool to predict possible alternative splicing events from Affymetrix probe set inputs. ASTALAVISTA [116] provides alternative splicing prediction for transcriptome data from GENCODE, REFSEQ, and ENSEMBL as well as from custom gene datasets. Furthermore, SpliceCenter [120] is a web server for predicting the influence of alternative splicing on RT-PCR, RNAi, microarray, and peptide-based data.

Both PolyA_DB [110] and AltTrans provide the information for alternative polyadenylation [111]. For AltTrans, the AltSplice pipeline on splicing and the AltPAS pipeline on polyadenylation were implemented. ASTD [126] also provides the variants for splicing, transcription initiation, and polyadenylation. Of note, the dataset of transcriptomics for alternative splicing is larger than for alternative polyadenylation. GRSDB [112] is a mammalian database of alternative splicing based on quadruplex forming G-rich sequences which modulate the 3′ end processing of pre-mRNAs.

Additionally, several comprehensive databases for alternative splicing have been developed such as HOLLYWOOD [113], ASD [114], BIPASS [117], ECgene [118], ASPicDB [121], AspAlt [125], H-DBAS [128], SPLOOCE [129], and APPRIS [130]. For example, the ECgene provides EST and serial analysis of gene expression (SAGE) data-based annotation and visualization for alternative splicing (AS). The ASPicDB provides EST-based tissue-specific splicing information of normal and cancer cells. The H-DBAS provides alternative splicing annotation based on RNA-Seq transcriptomics data. The APPRIS provides the annotation for principal isoform as the standard reference sequence for each gene.

Some resources of alternative splicing have special features such as splice signals in EuSplice [119], tandem splice sites in TassDB2 [127], mutational evidence-based analysis in Alternative Splicing Mutation Database [122], splicing proteins in SpliceAid 2 [131], and transcription factors in TFClass [132]. However, the impacts of alternative splicing on the spliced transcripts encoded protein structure are less addressed. Some databases such as ProSAS [123] and SpliVaP [124] also provide the protein isoforms from the alternative splicing effects. In ProSAS, the protein isoforms of splicing transcripts are annotated in Ensembl or SwissProt. In SpliVap, protein signatures of alternative forms are annotated in terms of Pfam domains and PRINTS fingerprints.

## 6. Conclusion

Accumulating evidence shows that alternative splicing can be selectively targeted in several genes of cancer cells. An exciting possibility raised by this study is that the effectiveness of anticancer therapies may be enhanced by clinical drugs, natural products, and their synthesized analogs that target alternative splicing machinery. Some alternative splicing-related databases and web servers may also helpful to improve the alternative splicing therapy for treating cancer and other diseases.

## Figures and Tables

**Table 1:** The bioinformatics resources related to alternative splicing (yrs 2001–2013)*.

AsMamDB [104]	An alternative splice database of mammals (website is unavailable)
PALS db [105]	Putative alternative splicing database
ASAP [106]	Alternative splicing annotation project (http://www.bioinformatics.ucla.edu/ASAP/)
EASED [107]	Extended alternatively spliced EST database
AVATAR [108]	Database for EST and mRNA
MAASE [109]	Alternative splicing database designed for splicing microarray (http://maase.genomics.purdue.edu/)
PolyA_DB [110]	Database for mRNA polyadenylation in mammalian
AltTrans [111]	Annotation for both alternative splicing and alternative polyadenylation
GRSDB [112]	Database of quadruplex forming G-rich sequences in alternative splicing sequences (http://bioinformatics.ramapo.edu/grsdb/)
HOLLYWOOD [113]	A comparative relational database of alternative splicing (http://hollywood.mit.edu/)
ASD [114]	A bioinformatics resource on alternative splicing
Splicy [115]	Prediction of alternative splicing from Affymetrix data
ASTALAVISTA [116]	Analysis of alternative splicing for custom datasets (http://genome.imim.es/astalavista/)
BIPASS [117]	Bioinformatics pipeline alternative splicing services
ECgene [118]	Alternative splicing database update (http://genome.ewha.ac.kr/ECgene/)
EuSplice [119]	A resource for splice signals and alternative splicing in eukaryotic genes (http://www.genome.com/products-1/integrated-genomics-resources/eusplice)
SpliceCenter [120]	A server for analysis of alternative splicing on RT-PCR, RNAi, microarray, and peptide-based studies (http://discover.nci.nih.gov/splicecenter/)
ASPicDB [121]	Database for alternative splicing analysis (http://www.caspur.it/ASPicDB/)
The alternative splicing mutation database [122]	A hub for analyzing alternative splicing from mutational evidence
ProSAS [123]	Database for analyzing alternative splicing in the context of protein structures (http://www.bio.ifi.lmu.de/ProSAS/)
Splice-mediated Variants of Proteins (SpliVaP) [124]	Signatures for protein isoforms due to alternative splicing (http://www.bioinformatica.crs4.org/tools/dbs/splivap/)
AspAlt [125]	A interdatabase for comparative analysis of alternative transcription and splicing (http://www.genome.com/products-1/integrated-genomics-resources/products-integrated-genomics-resources-igr-aspalt)
ASTD [126]	Alternative splicing and transcript diversity database
TassDB2 [127]	A comprehensive database of subtle alternative splicing (http://www.tassdb.info/)
H-DBAS [128]	Human-transcriptome database for alternative splicing (http://h-invitational.jp/h-dbas/)
SPLOOCE [129]	Analysis server of human splicing variants (http://www.bioinformatics-brazil.org/splooce/)
APPRIS [130]	Annotation of human alternative splice isoforms (http://appris.bioinfo.cnio.es/)
SpliceAid 2 [131]	Database of human splicing factors expression data and RNA target motifs (http://www.introni.it/spliceaid.html)
TFClass [132]	Classification of human transcription factors (http://tfclass.bioinf.med.uni-goettingen.de/)

*The websites for some resources without function currently are not provided.
